# Association between bedtime and female infertility: a secondary analysis from a cross-sectional study

**DOI:** 10.3389/fendo.2024.1340131

**Published:** 2024-06-20

**Authors:** Hanzhi Zhang, Jun Zhang, Wenxiu Chen, Hongyu Liu, Jingfei Chen, Jianlin Chen

**Affiliations:** ^1^ Reproductive Medicine Center, Department of Obstetrics and Gynecology, The Second Xiangya Hospital, Central South University, Changsha, Hunan, China; ^2^ Department of Obstetrics, Department of Obstetrics and Gynecology, The Second Xiangya Hospital, Central South University, Changsha, Hunan, China

**Keywords:** bedtime, infertility, lifestyle, non-linear relationship, cross-sectional studies

## Abstract

**Objective:**

To evaluate the association between bedtime and infertility and to identify the optimal bedtime for women of reproductive age.

**Methods:**

We conducted a cross-sectional study using data from 3,903 female participants in the National Health and Nutrition Examination Survey (NHANES) from 2015 to 2020. The effect of bedtime on female infertility was assessed using the binary logistic regression in different models, including crude model and adjusted models. To identify the non-linear correlation between bedtime and infertility, generalized additive models (GAM) were utilized. Subgroup analyses were conducted by age, body mass index (BMI), waist circumference, physical activity total time, marital status, smoking status, drinking status and sleep duration.

**Results:**

After adjusting for potential confounders (age, race, sleep duration, waist circumference, marital status, education, BMI, smoking status, drinking status and physical activity total time), a non-linear relationship was observed between bedtime and infertility, with the inflection point at 22:45. To the left side of the inflection point, no significant association was detected. However, to the right of it, bedtime was positively related to the infertility (OR: 1.22; 95% CI: 1.06 to 1.39; *P* = 0.0049). Subgroup analyses showed that late sleepers with higher BMI were more prone to infertility than those with a lower BMI (BMI: 25–30 kg/m^2^: OR: 1.26; 95% CI: 1.06 to 1.51; *P* = 0.0136; BMI ≥ 30 kg/m²: OR: 1.21, 95% CI: 1.09 to 1.34; *P* = 0.0014).

**Conclusion:**

Bedtime was non-linearly associated with infertility, which may provide guidance for sleep behavior in women of childbearing age.

## Introduction

Infertility is defined as the inability to achieve a clinical pregnancy following 12 months of regular and unprotected sexual intercourse (1). Infertility is thought to affect millions of individuals and couples worldwide. The global incidence of infertility ranges from 9% to 18%, with a continuous increase in recent years (2, 3). In the United States, up to 15% of couples are affected by infertility (4). Infertility represents a widespread global health concern, highlighting the significance of identifying risk factors and prioritizing preventive measures.

Reproduction is intricately controlled by a multitude of hormonal mechanisms, and any disruptions in these hormonal regulations may potentially result in infertility. It is widely recognized that sleep patterns and alterations in circadian rhythms significantly impact the secretion of various reproductive hormones (5, 6). The circadian clock exerts its effect through the hypothalamic-pituitary-gonadal (HPG) axis and plays a key role in the regulation of reproductive function (7). Disruptions in the circadian rhythm, often originating from sleep behaviors, can lead to irregularities in the secretion of reproductive hormones, ultimately affecting reproductive function. Hence, it is crucial to further investigate the effect of sleep behaviors on infertility.

Previous studies have investigated the relationship between sleep behaviors and infertility. For example, research has indicated that unhealthy sleep behaviors due to night shift work can adversely affect reproductive function (8–10), and individuals diagnosed with infertility tend to display a nocturnal chronotype (11). These findings indicate a potential association between bedtime and infertility. However, the research on their association is limited. A previous cross-sectional study investigated the association between bedtime and infertility and found a positive linear association (12). However, it remains unclear whether a nonlinear relationship exists between bedtime and infertility, or if there might be an optimal bedtime.

The fast-paced nature of modern society has resulted in significant alterations to people’s lifestyles, particularly with regard to their sleep patterns. Due to the demands of work and study, an increasing number of individuals tend to delay their bedtime. Therefore, our study aims to explore the relationship between bedtime, a modifiable sleep behavior, and infertility, and identify an optimal bedtime. These would provide valuable health guidance for women in their childbearing age. These findings hold the potential to make substantial contributions to improving public health outcomes. This analysis was conducted using data obtained from the National Health and Nutrition Examination Survey (NHANES) database (RRID: SCR_013201), covering a nationally representative cohort of women aged 18 to 44 years. The data spanned the years 2015 to 2020.

## Materials and methods

### Study population

**Figure 1:**
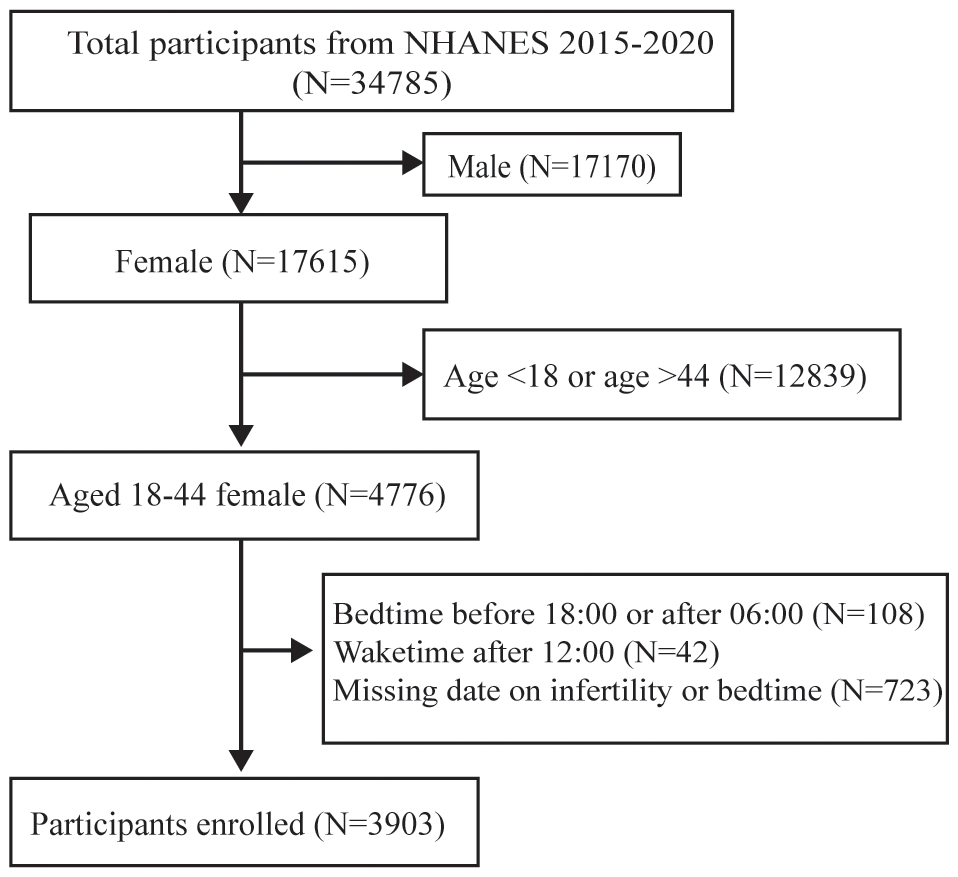
Flow chart for participants recruitment, NHANES 2015-2020.

NHANES is a national cross-sectional survey designed to evaluate the health and nutritional status of the United States. The data of this study were retrieved from three consecutive cycles (2015–2016, 2017–2018, and 2019–2020) of the NHANES. A total of 3903 participants were recruited and selected according to standard exclusion criteria, as follows: 1) Male (n = 17,170); 2) age < 18 or age >44 (n = 12,839); 3) Bedtime before 18:00 or after 06:00 (n = 108); 4) Waketime after 12:00 (n=42); 5) Missing data on infertility or bedtime (n= 723). An illustration of the participant selection process is depicted in **Figure 1**. The NHANES project information is gathered biannually in a six-month cycle. The two periods span November 1 through April 30 and from May 1 to October 31, thus covering all the months in the year. The frequency distribution graph for ‘The two periods’ is shown in **Supplementary Table S1**. All research methods of the NHANES were conducted in accordance with the Declaration of Helsinki. NHANES approved by National Center for Health Statistics (NCHS) Ethics Review Board, is a major project of the NCHS within the Centers for Disease Control and Prevention (CDC). Because the NHANES database is publicly accessible, no additional ethical approvals are required. The information and resources within can be used by other researchers to replicate or reproduce the study. Access to the research design and data is available at https://www.cdc.gov/nhcs/nhanes/.

### Main variables

Infertility is characterized by the inability to achieve conception following 12 months or longer of consistent and unprotected sexual intercourse, either due to diminished fertility of the individual or their partner. In our study, the evaluation of infertility status was derived from the NHANES Reproductive Health Questionnaire. Specifically, participants were asked the following inquiry, “Have you ever attempted to become pregnant over a period of at least a year without becoming pregnant?” (RHQ.074). Participants who responded with “yes” were classified as infertile.

Bedtime information was obtained from the NHANES Sleep Disorder Questionnaire, specifically through the query: “What time do you usually fall asleep on weekdays or workdays?” (SLQ.300).

### Covariates

Covariates were selected according to previous researches, including age (RIDAGEYR), BMI (BMXBMI), waist circumference (BMXWAIST), race (RIDRETH3), marital status (DMDMARTZ), education (DMQ.141), physical activity (PAQ), sleep duration (SLD.012), smoking status (SMQ.040), and drinking status (ALQ). Participants reporting alcohol consumption exceeding 0 g/week were categorized as drinkers. The overall quantity of physical activity was calculated by adding time spent at work (PAD.615 and PAD.630), walking/bicycling (PAD.645), and recreational activities (PAD.660 and PAD.675).

### Statistical analysis

The statistical analysis process was conducted in four steps. First, we examined the baseline characteristics of participants, who were categorized based on whether they had infertility or not, using a weighted sample. Continuous variables were expressed as mean ± standard deviation (SD), and categorical variables were expressed as percentages. Second, following the recommendation of the Strengthening the reporting ofobservational studies in epidemiology (STROBE) statement (13), logistic regression models were applied to estimate the independent correlation between bedtime and infertility before or after the adjustment of confounders. Third, the generalized additive models (GAM) were used to find the non-linear correlations between bedtime and infertility. Smooth curve fitting was performed to draw curves of the association after full adjustment. Bedtime is plotted on the X-axis, and the probability of infertility occurring is represented on the Y-axis. The probability is calculated using the formula P=1/(1+exp(-Y)). Red line represents the smooth curve fit between variables. Blue bands represent the 95% of confidence interval from the fit. And the piecewise linear regression model was used to find the threshold impact of the bedtime on infertility. Fourth, subgroup analyses were carried out with the assistance of stratified linear regression models, and changes and interactions between subgroups were identified through likelihood ratio tests.

Data analysis was performed using R (The R Foundation; http://www.r-project.org; version 4.2.0) and EmpowerStats (www.empowerstats.net, X&Y solutions, Inc. Boston, Massachusetts). A two-sided *P* value of less than 0.05 was considered to indicate statistical significance.

## Results

### The selection of participates

Among the 34,785 participants, 30,882 were not included in the analysis. Out of these, 17,170 were male, 12,839 were with age <18 or > 44, 108 with bedtime before 18:00 or after 06:00, 42 with waketime after 12:00, and 723 showed missing information on infertility or bedtime. Consequently, the study focused on a subset of 3,903 individuals for further investigation (**Figure 1**).

### Baseline characteristics of participants

**Table 1** illustrates the baseline characteristics of the participants who were selected for the study. There were 440 in the infertile group and 3463 in the fertile group. Participants with infertility had an older age (34.3 years vs. 30.5 years, *P* < 0.0001), higher BMI (31.7 vs 29.2, *P* = 0.0002), and higher waist circumference (102.1 vs. 94.5, *P* < 0.0001). As for the sleep-related variates, participants with infertility had significantly shorter sleep duration (7.9 hours vs. 7.6 hours, *P* = 0.0033). Additionally, individuals in infertility group were more likely to be smokers. However, there was no statistically significant difference in education, physical activity, race, and drinking status between fertile and infertile groups.

**Table 1:** Weighted demographic characteristics of selected participants from the NHANES 2015-2020.

Characteristic	Fertile	Infertile	P-value
Numbers of participants	3463	440	
Bedtime	22:49 (22:44, 22:53)	23:00 (22:48, 23:11)	0.1004
Sleep duration (hours)	7.9 (7.8, 7.9)	7.6 (7.5, 7.8)	0.0033
Age (years)	30.5 (30.2, 30.9)	34.3 (33.3, 35.2)	<0.0001
Height (cm)	162.5 (162.1, 162.9)	163.4 (162.6, 164.3)	0.0532
Weight (kg)	77.1 (75.9, 78.5)	84.8 (81.7, 87.8)	<0.0001
BMI (kg/m^2^)	29.2 (28.7 ,29.6)	31.7 (30.5 ,32.9)	0.0002
Waist circumference (cm)	94.5 (93.5 ,95.6)	102.13 (99.8 ,104.5)	<0.0001
Physical activity total time (%)			0.2954
<150 (min/week)	12.6 (11.0, 14.4)	15.1 (10.8, 20.8)	
≥150 (min/week)	87.4 (85.6, 89.0)	84.9 (79.2, 89.3)	
Education (%)			0.1510
Less than 9^th^ grade	3.5 (2.7, 4.6)	1.7 (0.8, 3.3)	
High school or equivalent	28.8 (25.8, 32.0)	32.0 (25.3, 39.6)	
Some college or over	67.7 (64.1, 71.1)	66.3 (58.6, 73.3)	
Race (%)			0.1894
Non-Hispanic Black	13.3 (11.0, 16.1)	10.6 (8.0, 14.0)	
Non-Hispanic White	54.6 (50.2, 58.9)	59.7 (53.4, 65.7)	
Mexican American	12.0 (9.5, 15.2)	11.9 (8.6, 16.4)	
Other Race	20.0 (17.6, 22.7)	17.8 (14.0, 22.2)	
Marital status (%)			<0.0001
Widowed/Divorced/ Separated/Never Married	43.3 (40.9, 45.8)	21.2 (17.3, 25.6)	
Married/Living with Partner	56.7 (54.2, 59.1)	78.8 (74.4, 82.7)	
Smokers (%)			<0.0001
Never	71.4 (68.6, 74.0)	56.2 (50.8, 61.6)	
Former/Now	28.6 (26.0, 31.4)	43.8 (38.5, 49.2)	
Drinkers (%)			0.5290
No	78.3 (76.1, 80.4)	76.3 (68.8, 82.4)	
Yes	21.7 (19.6, 23.9)	23.7 (17.6, 31.2)	

Data in the table: For continuous variables: survey-weighted mean (95% confidence interval), P-value was by survey-weighted linear regression (svyglm). For categorical variables: survey-weighted percentage (95% confidence interval), P-value was by survey-weighted Chi-square test (svytable).

BMI, body mass index.

### The relationship between bedtime and infertility

Weighted binary logistic regression models were employed to assess the correlation between bedtime and infertility (**Table 2**). In the crude model, bedtime didn’t show correlation with infertility (OR = 1.09, 95% confidence interval (CI): 0.99, 1.19, *P* = 0.0896). In the minimally adjusted model (adjusted age, race, sleep duration and waist circumference), bedtime had positive correlation with infertility (OR = 1.15, 95% CI: 1.05, 1.26, *P* = 0.0042). In the fully adjusted model, the association remained statistically significant (OR = 1.17, 95% CI: 1.07, 1.29, *P* = 0.0022). A similar trend was observed when bedtime was treated as a categorical variable in the sensitivity analysis (*p* for trend = 0.0104). Additionally, based on the fact that NHANES project information is gathered biannually in a six-month cycle, we included the examination period as an additional adjustment variable in Model III. As shown in **Supplementary Table S2**, the inclusion of this covariate did not result in significant changes to the effect estimates compared to Model II.

**Table 2:** Relationship between bedtime and infertility in different models.

Exposure	Crude Model		Model I		Model II	
	OR (95%CI)	P value	OR (95%CI)	P value	OR (95%CI)	P value
bedtime (continuous)	1.09 (0.99, 1.19)	0.0896	1.15 (1.05, 1.26)	0.0042	1.17 (1.07, 1.29)	0.0022
bedtime (categorical)						
18:00 - ≤ 21:00	Ref		Ref		Ref	
21:00 - ≤ 22:00	0.82 (0.50, 1.34)	0.4277	0.99 (0.62, 1.57)	0.9657	0.96 (0.59, 1.54)	0.8531
22:00 - ≤ 23:00	0.95 (0.59, 1.53)	0.8426	1.23 (0.82, 1.86)	0.3213	1.32 (0.87, 1.98)	0.1957
23:00 -- 06:00	1.13 (0.67, 1.89)	0.6488	1.57 (0.98, 2.49)	0.0655	1.66 (1.04, 2.65)	0.0422
P for trend		0.3191		0.0213		0.0104

Curde model adjust for None.

Model I adjusted for age; race; sleep duration; waist circumference.

Model II adjusted for age; race; sleep duration; waist circumference; marital status; education; BMI; smoking status; drinking status; physical activity total time.

OR, odds radio; CI, confidence interval; Ref: reference; BMI, body mass index.

### The analyses of non-linear relationship

**Figure 2:**
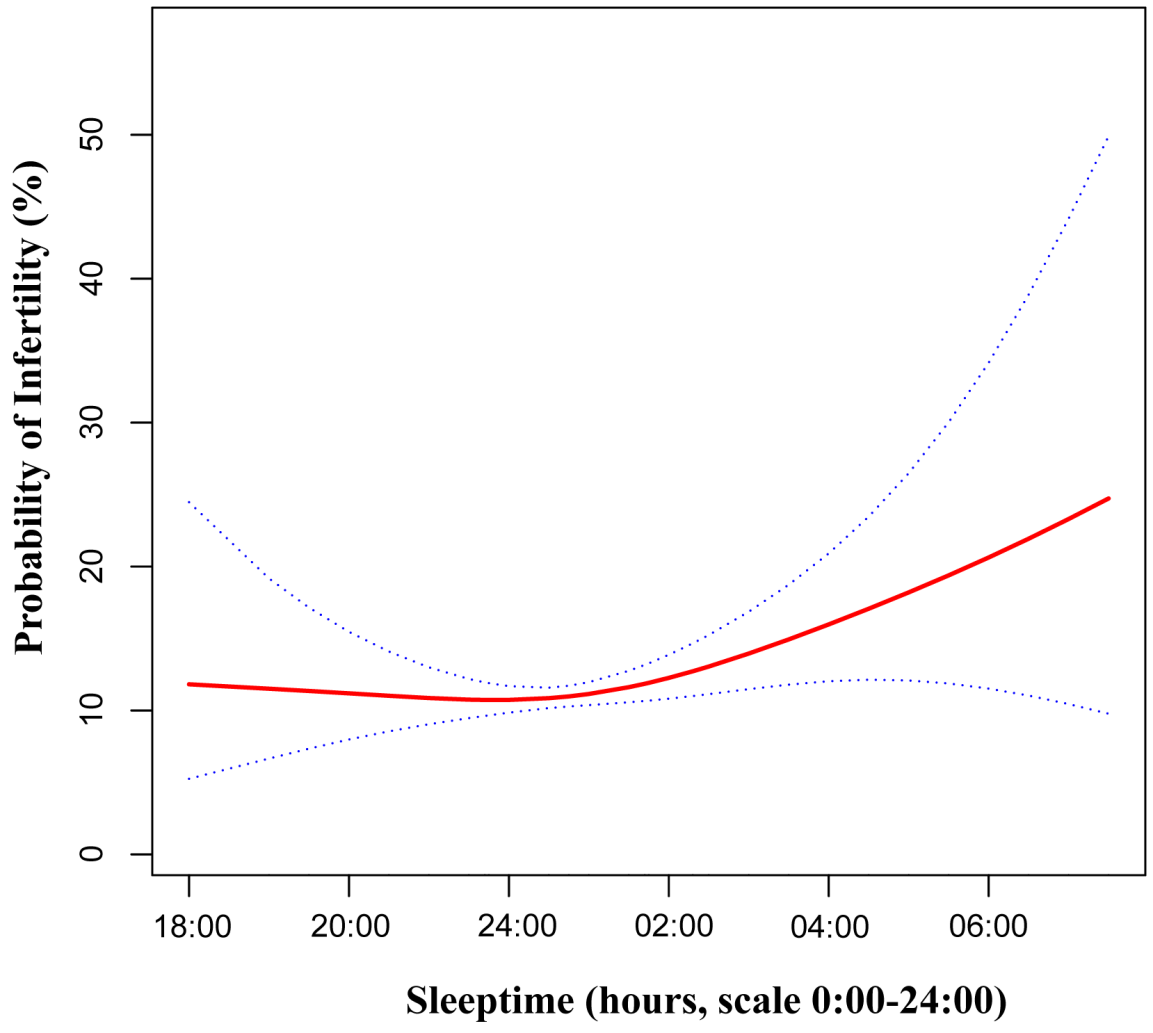
Adjusted associations of bedtime with female infertility. A non-linear relationship was found. Red line represents the smooth curve fit between variables. Blue bands represent the 95% of confidence interval from the fit. Adjusted:age, race, sleep duration, BMI, waist circumference, marital status, education, smoking status, drinking status and physical activity. BMI, body mass index.

To comprehensively examine the association between bedtime and infertility, a smoothing curve fitting was performed (**Figure 2**). We found non-linear relationship between bedtime and infertility, after adjusting age, race, sleep duration, BMI, waist circumference, marital status, education, smoking status, drinking status and physical activity. By employing a two-piecewise linear regression model, we were able to identify that the inflection point was situated at 22:45 (**Table 3**). To elucidate, on the left of the inflection point, the effect size, 95% CI and *P* value were 0.90, 0.74 to 1.09, 0.2839. However, on the right side of the inflection point, a clear positive correlation between bedtime and infertility was observed (OR: 1.22; 95% CI: 1.06 to 1.39; *P* = 0.0049). In sensitivity analyses that included participants with bedtime phases from 20:00 to 4:00, the non-linear relationship and inflection points remained stable (**Supplementary Figure S1**, **Supplementary Table S5**).

**Table 3:** Threshold Effect Analysis of bedtime and infertility using two-piecewise Linear Regression.

Inflection point of bedtime	Effect size (OR)	95%CI	P value
<22:45	0.90	0.74 to 1.09	0.2839
≥22:45	1.22	1.06 to 1.39	0.0049

Adjusted: age; race; sleep duration; waist circumference; marital status; education; BMI; smokers; drinkers; physical activity total time. OR, odds radio; CI, confidence interval; BMI, body mass index.

### The results of subgroup analyses

The outcomes of the subgroup analyses were detailed in **Table 4**. The test for interaction was significant for BMI (*P* for interaction = 0.0280), while the test for interactions involving age, waist circumference, physical activity, marital status, smoking status, drinking status and sleep duration didn’t reach statistically significance (*P* values for interactions were larger than 0.05). Similarly, the time period in which participants were included did not affect the relationship between bedtime and infertility (**Supplementary Table S3**). Based on these findings, BMI was identified as a potential confounding variable that could modify the association between bedtime and infertility. Significant differences in effect sizes of bedtime on infertility were observed across different BMI categories. Late sleepers with a higher BMI were found to be more likely to experience infertility compared to those with a lower BMI (BMI: 25–30 kg/m^2^: OR: 1.26; 95% CI: 1.06 to 1.51; *P* = 0.0136; BMI ≥ 30 kg/m²: OR: 1.21, 95% CI: 1.09 to 1.34; *P* = 0.0014).

**Table 4:** Effect size of bedtime on infertility in subgroups analysis.

Characteristic	OR (95%CI)	P value	P for interaction
Age (year)			0.0761
≤30	1.13 (0.98, 1.30)	0.0964	
30-40	1.25 (1.08, 1.45)	0.0059	
>40	0.98 (0.81, 1.18)	0.8127	
BMI (kg/m^2^)			0.0280
<25	0.99 (0.84, 1.17)	0.9283	
25-30	1.26 (1.06, 1.51)	0.0136	
≥30	1.21 (1.09, 1.34)	0.0014	
Waist circumference (cm)			0.7600
<80	1.13 (0.86, 1.49)	0.3781	
≥80	1.18 (1.07, 1.30)	0.0017	
Physical activity total time (min/week)			0.8694
<150	1.23 (0.94, 1.60)	0.1322	
≥150	1.15 (1.02, 1.30)	0.0252	
Marital status			0.7649
Widowed/Divorced/Separated/ Never Married	1.15 (0.97, 1.35)	0.1068	
Married/Living with Partner	1.18 (1.06, 1.31)	0.0036	
Smokers			0.0844
Never	1.12 (0.97, 1.29)	0.1408	
Former/Now	1.33 (1.16, 1.53)	0.0003	
Drinkers			0.0569
No	1.12 (1.02, 1.24)	0.0277	
Yes	1.38 (1.13, 1.69)	0.0030	
Sleep duration (hours)			0.6677
<7	1.16 (0.97, 1.38)	0.1095	
7-8	1.09 (0.89, 1.33)	0.3998	
8-9	1.29 (1.06, 1.58)	0.0154	
≥9	1.14 (0.97, 1.35)	0.1320	

Adjusted: age; race; sleep duration; waist circumference; marital status; education; BMI; smokers; drinkers; physical activity total time. OR, odds radio; CI, confidence interval; BMI, body mass index

In each case, the model is not adjusted for the stratification variable.

## Discussion

The impact of daily lifestyle factors, such as sleep and diet, on health and biological aging, and their relationship to infertility, has been studied in the field of reproductive medicine and health sciences. Previous research has provided evidence of the significant influence of sleep and diet on biological aging and their association with infertility. For instance, Ruijie Xie et al. demonstrated that a pro-infammatory dietary pattern is associated with biological aging (14). Xu Gao and colleagues elucidated that better sleep quality can mitigate accelerated biological aging caused by air pollution (15).

In line with prior studies, a previous cross-sectional study involving 227 participants reported a higher proportion of individuals with an evening chronotype among those with infertility (11). Furthermore, another cross-sectional study comprising 2,175 participants showed a linear positive association between bedtime and infertility (12). Building upon these previous studies, our research explored in-depth by investigating the non-linear relationship between bedtime and infertility, as evidenced by **Supplementary Table S4**.

It’s worth noting that the previous study did not account for waist circumference and alcohol consumption as potential confounders. Waist circumference serves as an anthropometric measure used to evaluate central obesity (16). Obesity has been recognized as a risk factor contributing to infertility (17, 18). Similarly, with regards to alcohol consumption, research has indicated that it acts as a predictor for infertility in women (19). The consumption of alcohol was linked to an elevated risk of experiencing infertility (20). Hence, in the present study, we introduced waist circumference and drinking status as additional confounders to be considered in the adjustment process, and we found that the association between bedtime and infertility persisted.

Furthermore, our study has taken a step further by investigating the non-linear relationship between bedtime and infertility (**Table 3**). An inflection point was observed at 22:45. Prior to this inflection point, the association was not statistically significant. However, on the right side of the inflection point, a significant increase in infertility incidence was observed with a delay in bedtime. This novel finding provides valuable guidance on the ideal bedtime for women of childbearing age, potentially contributing to the improvement of their reproductive health.

Regarding the relationship between bedtime and infertility, we suggest the following mechanisms. Participants with later bedtimes were more exposed to light during the night, which may suppress melatonin production and lead to disruption of circadian rhythms (21). On the one hand, melatonin acts as a free radical scavenger, typically protecting oocytes from oxidative stress. Decreased melatonin secretion may lead to increased vulnerability of oocyte to oxidative stress injury (22). On the other hand, the disruption of circadian rhythm could affect the secretion of HPG axis-related hormones through the suprachiasmatic nucleus (SCN). Circadian rhythms regulate the rhythmic behaviors and physiological fluctuations observed in various species. In mammals, these coordinated oscillations are mediated by the SCN (23). The SCN is closely related to the secretion of gonadotropin-releasing hormone (GnRH) neurons and a variety of reproductive hormones (24). One pathway comprises direct innervation of GnRH neurons by the SCN (25, 26), while the other involves an indirect circuitry where the SCN communicates with the HPG axis via Kisspeptin neurons (27, 28). Kisspeptin neurons located in the anterior ventral periventricular area play a pivotal role in triggering the luteinizing hormone (LH) surge. Concurrently, another subset of hypothalamic kisspeptin neurons in the arcuate nucleus exerts control over the HPG axis by regulating GnRH release (29). Collectively, potential alterations in the secretion of these hormones among late sleepers could explain the elevated risk of infertility.

In the subgroup analyses performed in this study, stratification variables included age, smoking status, BMI, marital status, total physical activity time, waist circumference, drinking status, and sleep duration. We found a positive association between bedtime and infertility among participants with a BMI equal to or greater than 25 kg/m^2^. However, this relationship was not significant among participants with a BMI less than 25 kg/m^2^. The plausible explanation for this phenomenon is that the obesity could influence the secretion of HPG axis associated hormones, thereby affecting menstrual cycle and ovulation (30, 31). The disruptive effects of circadian changes could be viewed as an added challenge to the secretion of these hormones. Individuals with a high BMI are at an elevated risk of experiencing infertility due to this effect. This may provide a potential explanation for why later bedtimes are more likely to result in infertility among populations with higher BMI levels.

Our study has several significant strengths. First, we utilized data obtained from the NHANES database, well-known for its comprehensive coverage and representative nature. Second, our investigation revealed a non-linear link between bedtime and infertility. Third, the utilization of threshold effect analysis resulted in the identification of an inflection point at 22:45, providing valuable advice regarding optimal bedtime for women of reproductive age. Fourth, we conducted subgroup analyses that revealed the impact of BMI on the correlation between bedtime and infertility.

Nonetheless, our study is accompanied by certain limitations. First, despite revealing the association between bedtime and infertility, establishing causation is not feasible due to the cross-sectional nature of the study. Prospective studies are necessary to explore the causal relationship between bedtime and female infertility in the future. Second, our study relied on self-reported data, which introduces potential recall bias. Third, even though we extensively adjusted for confounding factors, cross-sectional studies may not completely capture full complexity of relationships between variables and can be susceptible to selection bias and unaccounted-for confounders which were not included or recorded in the NHANES. This could potentially influence our results. Fourth, infertility status was self-reported by women, and information related to the fertility condition of the partner was not included in the NHANES. Fifth, the dataset originates from a nationwide U.S. survey, thus requiring further validation for its applicability across diverse ethnic groups.

In conclusion, our research findings suggest a non-linear relationship between bedtime and infertility, and have identified an optimal bedtime. This provides valuable health guidance for women of childbearing age.

## Data availability statement

Publicly available datasets were analyzed in this study. This data can be found here: https://www.cdc.gov/nchs/nhanes/index.htm.

## Ethics statement

All data obtained from NHANES, which was reviewed and approved by National Center for Health Statistics (NCHS) Ethics Review Board and all subjects agreed on the survey and signed written consent. The NHANES was conducted in accordance with local legislation and institutional requirements. Because the NHANES database is publicly accessible, no additional ethical approvals are required. The studies were conducted in accordance with the local legislation and institutional requirements. The participants provided their written informed consent to participate in this study.

## Author contributions

HZ: Data curation, Software, Visualization, Writing – original draft. JZ: Data curation, Writing – review & editing. WC: Formal analysis, Validation, Writing – original draft. HL: Data curation, Validation, Writing – original draft. JFC: Conceptualization, Investigation, Supervision, Writing – review & editing. JLC: Funding acquisition, Project administration, Supervision, Writing – review & editing.
