# Supplementary material for: Association between bedtime and female infertility: a secondary analysis from a cross-sectional study
Source: Front Endocrinol (Lausanne). 2024 Jun 20;15:1340131. doi: 10.3389/fendo.2024.1340131 (PMC11222632; doi:10.3389/fendo.2024.1340131)
Supplement: Supplementary file 1 [file DataSheet_1.docx]

Supplementary Material

Association between bedtime and female infertility: a secondary analysis from a cross-sectional study

**Hanzhi Zhang^#^, Jun Zhang^#^, Wenxiu Chen, Hongyu Liu, Jingfei Chen^*^, Jianlin Chen^*^**

^#^These authors contributed equally.

*** Correspondence:**Jingfei Chen: [jingfeichen@csu.edu.cn](mailto:jingfeichen@csu.edu.cn)Jianlin Chen: [jianlinchen@csu.edu.cn](mailto:jianlinchen@csu.edu.cn)

# Supplementary Figures and Tables

## Supplementary Figures


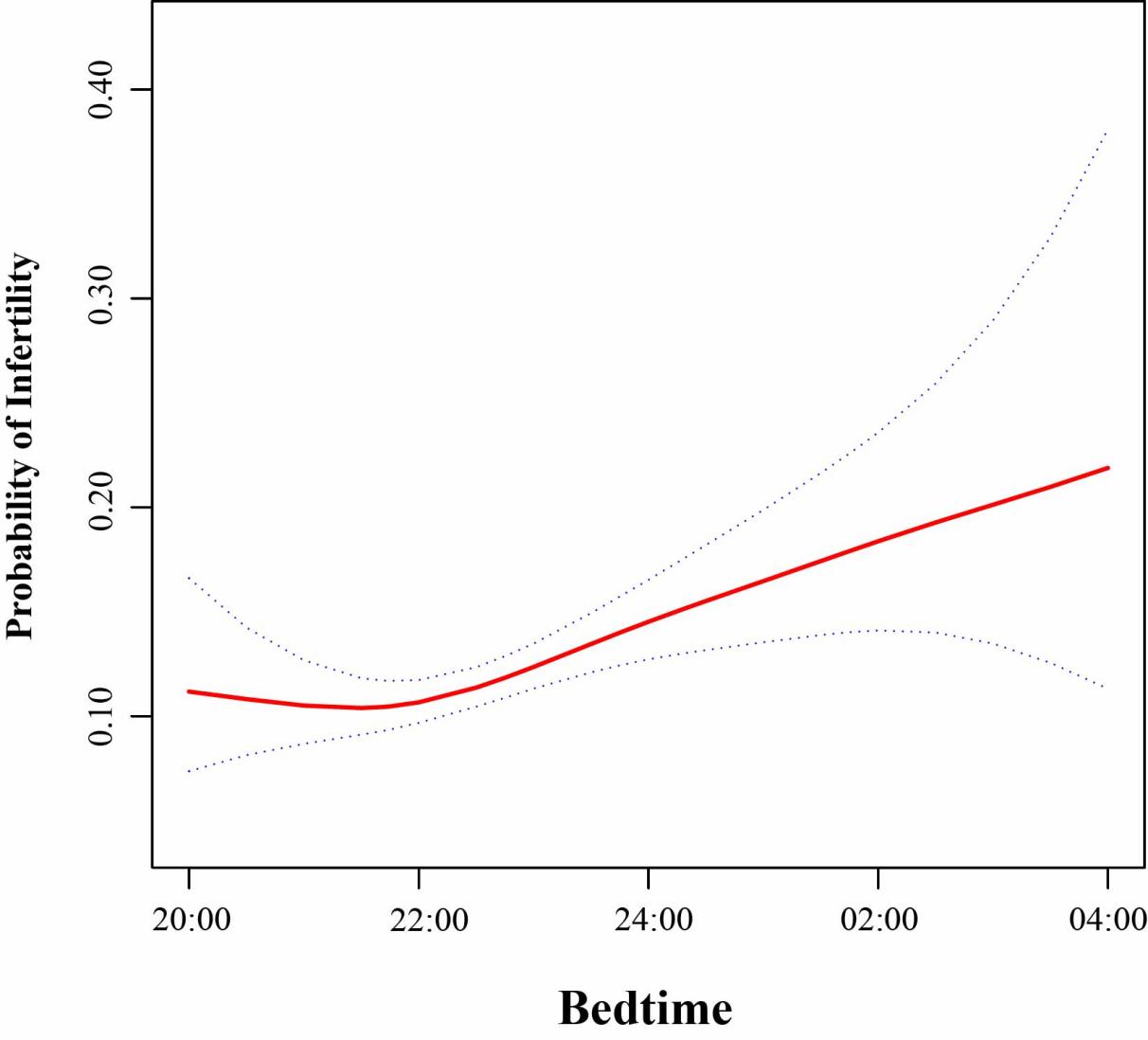


**Supplementary Figure 1.** A smooth curve fitting for the association between bedtime and infertility (Participants with bedtime 20:00-04:00). Adjusted: age; race; sleep duration; waist circumference; marital status; education; BMI; smokers; drinkers; physical activity total time. BMI, body mass index.

## Supplementary Tables

**Table S1. Table of frequency distribution of participants in two periods.**

| Periods | Total number of participants | Fertile | Infertile |
| --- | --- | --- | --- |
| November 1 through April 30 | 1953 | 1734 (50.1%) | 219 (49.8%) |
| May 1 through October 31 | 1950 | 1729 (49.9%) | 221 (50.2%) |

**Table S2 Relationship between bedtime and infertility in different models.**


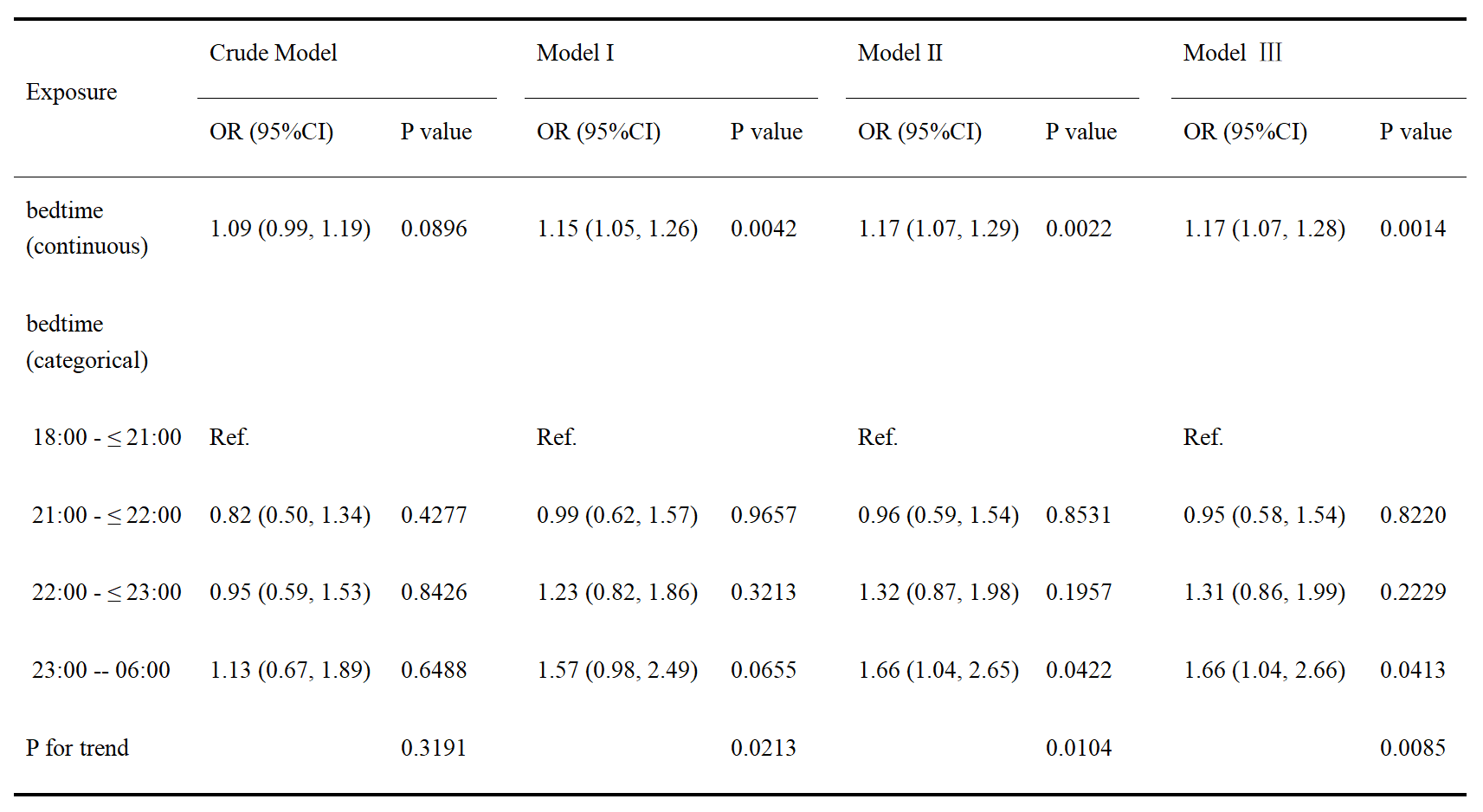


Curde model adjust for None.

Model Ⅰ adjusted for age; race; sleep duration; waist circumference.

Model Ⅱ adjusted for age; race; sleep duration; waist circumference; marital status; education; BMI; smoking status; drinking status; physical activity total time.

Model Ⅲ adjusted for age; race; sleep duration; waist circumference; marital status; education; BMI; smoking status; drinking status; physical activity total time; exam month.

OR, odds radio; CI, confidence interval; Ref: reference; BMI, body mass index.

**Table S3. Effect size of seasonal changes on the relationship between bedtime and infertility**

| Characteristic | OR (95%CI) | *P* value | *P* for interaction |
| --- | --- | --- | --- |
| Periods |  |  | 0.3278 |
| November 1 through April 30 | 1.12 (0.97, 1.29) | 0.1032 |  |
| May 1 through October 31 | 1.22 (1.10, 1.34) | 0.0057 |  |

Adjusted: age; race; sleep duration; waist circumference; marital status; education; BMI; smokers; drinkers; physical activity total time. OR, odds radio; CI, confidence interval; BMI, body mass index

**Table S4 Comparison with similar studies exploring the association between bedtime and infertility.**


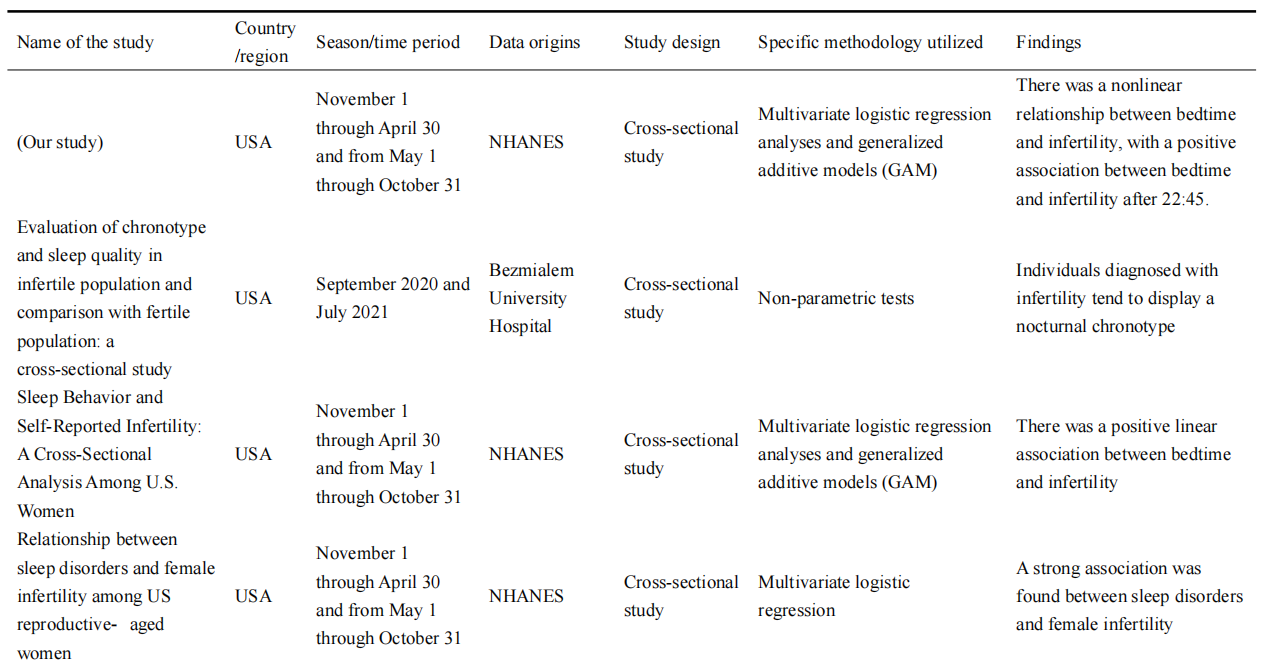

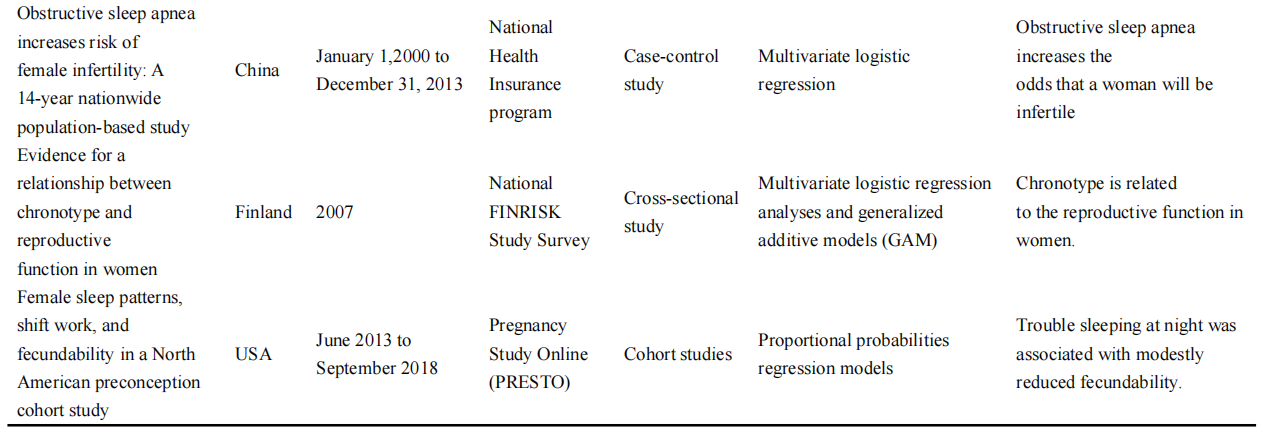


**Table S5 Sensitivity analysis of threshold effects in participants with different bedtime.**

| Participants' bedtime periods | Inflection point | Effect size (OR) | 95% CI | P value |
| --- | --- | --- | --- | --- |
| 18:00 - 6:00 |  |  |  |  |
|  | <22:45 | 0.90 | 0.74 to 1.09 | 0.2839 |
|  | ≥22:45 | 1.22 | 1.06 to 1.39 | 0.0049 |
| 18:00 - 4:00 |  |  |  |  |
|  | <22:45 | 0.92 | 0.75 to 1.11 | 0.3728 |
|  | ≥22:45 | 1.22 | 1.05 to 1.40 | 0.0069 |
| 20:00 - 6:00 |  |  |  |  |
|  | <22:45 | 0.86 | 0.70 to 1.07 | 0.1855 |
|  | ≥22:45 | 1.25 | 1.10 to 1.43 | 0.0010 |
| 20:00 - 4:00 |  |  |  |  |
|  | <22:45 | 0.87 | 0.70 to 1.08 | 0.2099 |
|  | ≥22:45 | 1.23 | 1.07 to 1.42 | 0.0045 |
